# Methoxy-Group Control of Helical Pitch in Stereoregular Poly(2-ethynylmethoxynaphthalene) Prepared by Rhodium Complex Catalyst

**DOI:** 10.3390/polym11010094

**Published:** 2019-01-08

**Authors:** Yasuteru Mawatari, Yoshiaki Yoshida, Kai Huang, Masayoshi Tabata

**Affiliations:** 1 Graduate School of Engineering, Muroran Institute of Technology, 27-1 Mizumoto-cho, Muroran 050-8585, Hokkaido, Japan; yyoshida@moleng.fuk.kindai.ac.jp; 2 Research Center for Environmentally Friendly Materials Muroran Institute of Technology, 27-1 Mizumoto-cho, Muroran 050-8585, Hokkaido, Japan; 3 Graphene New Materials Technology, JunFeng Business BuildingA-4F, Chongqing Road, Baoan District, Shenzhen 518101, China; kai.huang@granmtech.com; 4 Center of Environmental Science and Disaster Mitigation for Advanced Research, Muroran Institute of Technology, 27-1 Mizumoto-cho, Muroran 050-8585, Hokkaido, Japan; 5 Faculty of Science and Technology, Department of Applied Chemistry and Bioscience, Chitose Institute of Science and Technology, Bibi 65-758, Chitose 066-8655, Hokkaido, Japan

**Keywords:** conjugated polymer, polyacetylene, Rh-complex catalyst, helix, helical pitch, color

## Abstract

The position of the methoxy group in a poly(*n*-methoxy-2-ethynylnaphthalene) (PnMeO2EN) was found to control the helical pitch of the π-conjugated polymer in the solid state. These PnMeO2ENs were stereoregularly synthesized using an Rh-complex catalyst in ethanol or toluene as the solvent. The helical structure in the solid phase was confirmed by conventional analytical methods, namely diffuse reflective ultraviolet–visible light (UV–Vis) and Raman spectroscopies, X-ray diffraction, and ^13^C cross-polarization magic angle spinning NMR spectroscopy, together with molecular mechanics calculations, because the as obtained polymers were insoluble in common solvents. The color of poly(6-methoxy-2-ethynylnaphthalene) (P6MeO2EN) (yellow or red) depended on the polymerization solvent, whereas no such dependency was observed for the yellow-colored P7MeO2EN and P8MeO2EN. The helical structures energetically optimized by molecular mechanics indicate that the red- and yellow-colored P6MeO2ENs form contracted and stretched helices, respectively. Due to the relatively unconstrained rotations of the 6-methoxynaphthyl moieties, the methoxy groups in P6MeO2EN are less sterically hindered along the helical axis. On the contrary, P7MeO2EN and P8MeO2EN have stretched helices due to the considerable steric hindrance imparted by their methoxy groups. The thermal *cis*-to-*trans* isomerization of P6MeO2EN in the contracted-helix form required a somewhat higher temperature than that of the stretched helix.

## 1. Introduction

The unique shape and functionality of helical-structured polymers and supramolecules make them the object of keen research interest. Thus, numerous reports and reviews recently focused on the helix sense of such helical structures, describing methods for inducing/confirming the formation of one-handed helices, dynamic helix inversion caused by external stimuli, and sergeant/soldier roles for sensing chirality, ions, and molecular-size recognition [1,2,3]. In contrast, despite its importance, control of the helical pitch received relatively little attention. In view of the fact that the change of helical pitch, i.e., the mutual interconversion of stretched and contracted helices, is the basis of molecular spring operation, a better understanding of the corresponding factors of influence (e.g., dynamic changes of interactions between helical hosts and guest molecules) is expected to facilitate the preparation of novel functional materials [4,5].

Poly(phenylacetylene) derivatives (PPAs) are popular π-conjugated helical polymers that attracted considerable attention in the last two decades [6,7,8,9,10,11,12,13,14,15]. PPAs with highly stereoregular head-to-tail sequences and high *cis*-isomer contents are commonly synthesized using the [Rh(norbornadiene)Cl]_2_–NEt_3_ catalyst system under mild conditions [16,17]. Recently, helical PPAs were applied as chirality sensors [18,19], chiral stationary phases with changeable elution order [20], and nanowires for charge transport [21]. We reported the selective preparation of PPA and its derivatives with different helical pitches, i.e., with stretched and contracted helices, and described the drastic color changes accompanying the stretched-to-contracted transition [22,23,24,25]. The above transition was shown to smoothly occur upon heat treatment [22] and contact with appropriate solvents [24,25]. Moreover, poly(alkyl propiolate)s, which are aliphatic-substituted helical polyacetylenes, were demonstrated to undergo reversible stretched-to-contracted helix interconversion as a result of chemical exchange in solution [26,27].

PPAs form helical structures only when their main chain has a *cis*-configuration. However, the *cis*-to-*trans* isomerization is easily induced by external stimuli such as heat treatment [28], compression [29,30,31], and irradiation [32,33], which is accompanied by the collapse of the helical structure. Therefore, practical applications of helical molecules necessitate the development of robust helical structures. Previously, the helical pitch of poly(2-ethynylnaphthalene) (P2EN) was shown to be affected by the immersion of polymer into solvent, e.g., the yellow-colored stretched-helix P2EN that was synthesized in ethanol (EtOH) solvent was converted to red-colored contracted-helix P2EN upon immersion into toluene at room temperature [25]. Notably, the contracted-helix P2EN showed higher thermal stability than PPAs because of the strong π-stacking interactions between neighboring naphthyl rings in its side chain. Therefore, P2ENs are thought to be better heat-resistant helical π-conjugated polymers than other candidates.

**Scheme 1:**
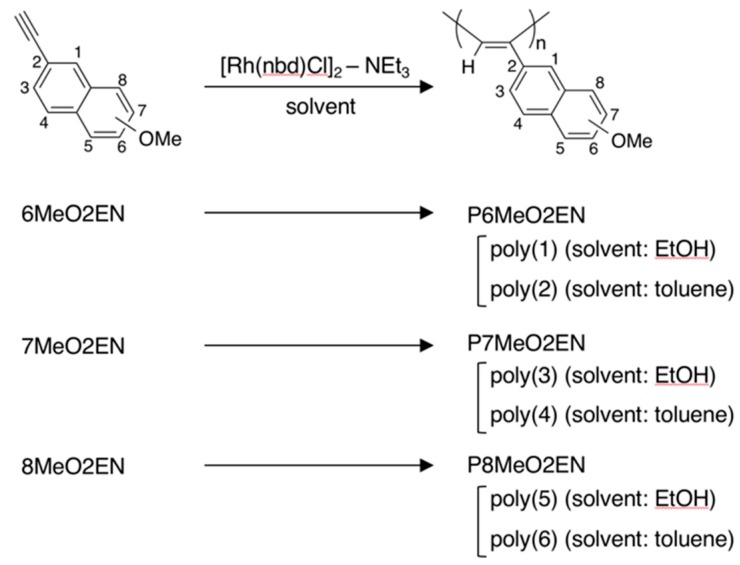
[Rh(norbornadiene)Cl]_2_–NEt_3_-catalyzed polymerization of *n*-methoxy-2-ethynylnaphthalenes (nMeO2ENs; *n* = 6, 7, 8).

The unique functions and properties of helical polymers can be enhanced by the introduction of various functional groups onto their side chain.s Reports on various applications of helical substituted polyacetylenes recently increased. However, these reports still do not have enough discussion on, e.g., how helical pitch affects the polymer properties, except for a few reports [13]. The introduction of functional groups onto the naphthyl rings in P2EN side chains is of great interest because these polymers need to be modified by the introduction of functional groups into the side chain in order to enhance and change the original chemical properties. Additionally, only a few studies of stereoregular P2EN and its derivatives prepared using the above Rh-complex-based catalyst system were reported [34], and the relationship between the position of the introduced functional group and the helical pitch of P2ENs is unfortunately still unclear, although there were many reports on their relationship in the case of PPA derivatives [35]. We believe that these detailed studies regarding the effect of substituent position on the helical pitch of aromatic polyacetylenes will contribute to the molecular design of helical structures of not only poly(arylacetylene)s, but also other helical polymers. Therefore, to bridge this gap and to enhance P2EN functionality, we herein report the synthesis of P2ENs bearing methoxy groups at positions 6, 7, and 8 of the naphthyl ring, together with characterization of the helical structure of the obtained polymers (Scheme 1). This work may be expected to facilitate the development of novel materials based on controlled helical structures, for instance, super-small electronics, highly sensitive sensors, drug delivery agents, and nanomachines.

## 2. Materials and Methods

### 2.1. Measurements

The ^1^H (500 MHz) and ^13^C NMR (125 MHz) spectra in solution were measured on a JEOL ECA-500 in CDCl_3_ at room temperature. The solid-state ^13^C cross-polarization/magic angle spinning (CPMAS) NMR (125 MHz) spectra were also measured on the same spectrometer using adamantine as the standard and 5 ms of contact time at room temperature. The diffuse reflective ultraviolet–visible light (DRUV–Vis) spectra of the polymers were recorded on a JASCO V570 spectrophotometer equipped with an ISV-470 integrating sphere accessory. The resonance Raman spectra of the polymers were recorded on a RENISHAW inVia Raman Microscope using laser light at 532 nm. Wide-angle X-ray scattering (WAXS) patterns of the polymers were recorded on a RIGAKU RINT Rapid II with CoKα (λ = 1.789 Å) as a radiation source. Differential scanning calorimetry (DSC) was performed on a SHIMADZU DSC-60, and traces were run in an atmosphere of N_2_ at a heating rate of 10 °C/min.

### 2.2. Materials

#### 2.2.1. General

Chemicals used for synthesis of all monomers were purchased from Tokyo Chemical Industry Co., Ltd., Japan and Junsei Chemical Co, Ltd., Japan. The polymerization catalyst [Rh(nbd)Cl]_2_ (2,5-norbornadiene rhodium(I) chloride dimer, Wako Chemical, Ltd., Japan) was used as received.

#### 2.2.2. Synthesis of Monomers

A monomer, 6-methoxy-2-ethynylnaphthalene (6MeO2EN), was prepared as described in the literature [36]. The monomers, 7-methoxy-2-ethynylnaphthalene (7MeO2EN) and 8-methoxy-2-ethynylnaphthalene (8MeO2EN), were prepared as described in Scheme S1 (Supplementary Materials) and purified by column chromatography on silica gel with *n*-hexane prior to use. ^13^C NMR spectra of three monomers are described in Figure S1 (Supplementary Materials) to compare with the ^13^C CPMAS spectra of polymers obtained.

- 6MeO2EN: pale yellow solid. ^1^H NMR (500 MHz, CDCl_3_): δ 7.94 (s, 1H, Ar), 7.69 (d, *J* = 9.0 Hz, 1H, Ar), 7.66 (d, *J* = 8.5 Hz, 1H, Ar), 7.49 (dd, *J* = 8.3 Hz, 1.8 Hz, 1H, Ar), 7.15 (dd, *J* = 9.0 Hz, 2.5 Hz, 1H, Ar), 7.10 (d, *J* = 2.5 Hz, 1H, Ar), 3.92 (s, 3H, O–CH_3_), 3.10 (s, 1H, –C≡C–H); ^13^C NMR (125 MHz, CDCl_3_): δ 158.44, 134.37, 132.07, 129.31, 129.14, 128.28, 126.80, 119.48, 116.91, 105.75, 84.18, 76.68, 55.32.
- 7MeO2EN: pale yellow solid. ^1^H NMR (500 MHz, CDCl_3_): δ 7.91 (s, 1H, Ar), 7.71 (d, *J* = 2.0 Hz, 1H, Ar), 7.69 (d, *J* = 1.5 Hz, 1H, Ar), 7.39 (dd, *J* = 8.3 Hz, 1.8 Hz, 1H, Ar), 7.16 (dd, *J* = 8.8 Hz, 2.8 Hz, 1H, Ar), 7.07 (d, *J* = 2.5 Hz, 1H, Ar), 3.91 (s, 3H, O–CH_3_), 3.14 (s, 1H, –C≡C–H); ^13^C NMR (125 MHz, CDCl_3_): δ 158.10, 134.02, 131.05, 129.22, 128.56, 127.73, 126.40, 119.82, 119.75, 105.52, 84.14, 77.29, 55.32.
- 8MeO2EN: pale yellow solid. ^1^H NMR (500 MHz, CDCl_3_): δ 8.45 (s, 1H, Ar), 7.72 (dd, *J* = 8.5 Hz, 0.5 Hz, 1H, Ar), 7.52 (dd, *J* = 8.5 Hz, 1.5 Hz, 1H, Ar), 7.42–7.35 (m, 2H, Ar), 6.81 (dd, *J* = 6.5 Hz, 2.0 Hz, 1H, Ar), 3.98 (s, 3H, O–CH_3_), 3.12 (s, 1H, –C≡C–H); ^13^C NMR (125 MHz, CDCl_3_): δ 155.13, 140.00, 129.05, 127.54, 127.10, 126.78, 125.05, 119.94, 118.55, 104.46, 84.37, 76.95, 55.53.

### 2.3. Polymerization Procedure

The three monomers (6MeO2EN, 7MeO2EN, and 8MeO2EN) were stereoregularly polymerized using the [Rh(nbd)Cl]_2_ catalyst with NEt_3_ as the cocatalyst in the polymerization solvent to afford corresponding polymers (Scheme 1). In a typical procedure, 2.3 mmol of monomer, 2.3 × 10^−2^ mmol of [Rh(nbd)Cl]_2_, and 2.3 mmol of NEt_3_ were dissolved in 11.4 mL of EtOH in a specially designed U-shaped flask [37]. The polymerization was carried out at 20 °C for 0.5 h and quenched by adding 200 mL of methanol. The as obtained polymer was filtered and washed with methanol, followed by vacuum drying at 4 × 10^−2^ torr at 40 °C for 24 h. All polymers were insoluble to any organic solvents at room temperature.

### 2.4. Computation

The energetically optimal conformations of the 20-mer of the 2EN unit having a methoxy group were determined using molecular mechanics (MM) calculations with the MMFF94 force field program [38] (Spartan’16 Windows version 1.1.0, Wavefunction, Inc., Irvine, CA, USA).

## 3. Results and Discussion

### 3.1. Synthesis of MeO-Substituted P2ENs (PnMeO2ENs)

The utilized monomers were selected based on the considerations below. The ethynyl group was introduced at the 2-position of the naphthyl ring, since Rh-catalyzed polymerization of 2-ethynylnaphthalene (2EN) was previously shown to afford highly *cis* helical P2EN. Moreover, Rh-catalyzed polymerization of *ortho*-substituted phenylacetylenes to afford PPAs is known to induce partial *cis*-to-*trans* isomerization and, thus, result in helical structure destruction [39], since 1-ethynylnaphthalene (1EN) monomers can be regarded as equivalents of *ortho*-substituted phenylacetylene monomers [40,41].

Considering the substituent, the MeO group was chosen owing to its small size and ease of introduction, and positions 6, 7, and 8 were chosen based on the considerations of raw material availability and ease of synthesis. Table 1 shows the results of Rh-catalyzed polymerization of nMeO2ENs, demonstrating that all reactions proceeded in high yields. All polymerization reactions were initiated by mixing monomer and catalyst solutions, and the obtained polymers precipitated as powders. In all cases, polymer yields in toluene exceeded those in EtOH. The use of gel permeation chromatography to determine the molecular weights of as-prepared polymers proved to be impossible because of their insolubility in common solvents such as toluene, chloroform, tetrahydrofuran, dimethylformamide, acetone, and alcohols.

**Figure 1:**
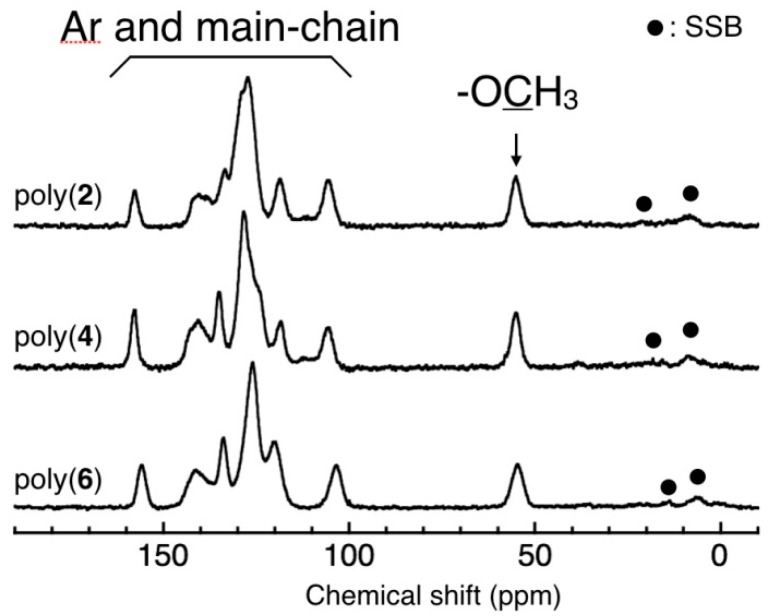
Room temperature ^13^C cross-polarization/magic angle spinning (CPMAS) NMR spectra of poly(**2**), poly(**4**), and poly(**6**). SSB represents a spinning side band.

The occurrence of polymerization was confirmed by ^13^C CPMAS NMR spectroscopy. Figure 1 shows the spectra of poly(**2**), poly(**4**), and poly(**6**), which were almost identical to those of poly(**1**), poly(**3**), and poly(**5**) (Figure S2, Supplementary Materials). No peaks assignable to monomer C≡C carbons (Figure S1, Supplementary Materials) were observed at 85–75 ppm in all spectra, which confirmed that polymerization afforded substituted polyacetylenes not containing any residual C≡C groups. Unfortunately, main-chain C=C peaks were not separated from aromatic C=C ones, although peaks observed at ~55 ppm could be assigned to the carbon in the MeO group.

### 3.2. Difference in Color of Polymer Powders

**Figure 2:**
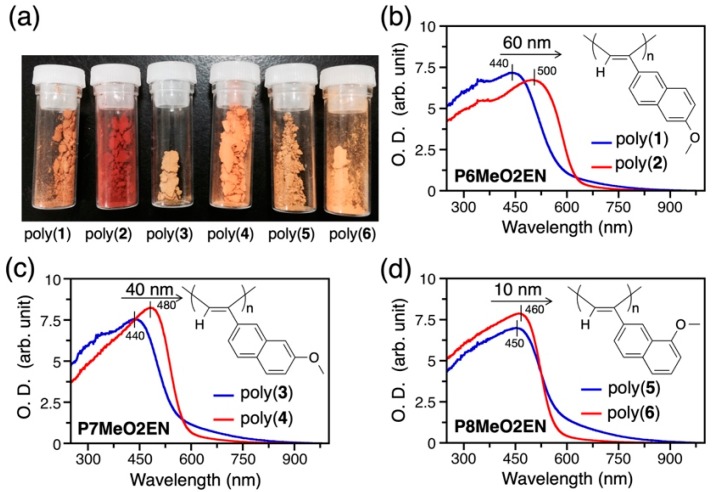
Photographs of poly(**1**–**6**) (**a**), diffuse reflectance ultraviolet–visible light (UV–Vis) spectra of poly(6-methoxy-2-ethynylnaphthalene)s (P6MeO2ENs) (**b**), P7MeO2ENs (**c**), and P8MeO2ENs (**d**).

Interestingly, the color of as prepared polymer powders depended on the position of the MeO group in the corresponding 2EN monomers, i.e., whereas all polymers prepared in EtOH (poly(**1**), poly(**3**), and poly(**5**)) were yellow, those prepared in toluene were red (poly(**2**)), orange (poly(**4**)), and yellow (poly(**6**)) (Figure 2a). Figure 2b–d show the diffuse reflective UV–Vis spectra of all polymers, revealing that the absorption maxima (*λ*_max_) of yellow polymers (poly(**1**), poly(**3**), poly(**5**), and poly(**6**)) were observed at ~450 nm. Conversely, the *λ*_max_ values of poly(**2**) and poly(**4**) were observed at longer wavelengths (500 and 480 nm, respectively).

Previously, we revealed that the color of substituted polyacetylenes is affected by the primary (*cis*/*trans*) and secondary (helical) structures of their main chain. The former factor influences the length of π-conjugation in the main chain, e.g., substituted polyacetylenes prepared using the Rh-complex-based catalyst are normally yellow, having a *cis-transoidal* (*ct*) main chain with relatively short-range π-conjugation (over two or three monomer units) [42]. In the case when the main chain contains the *trans-transoidal* (*tt*) isomer (e.g., as a result of *cis*-to-*trans* isomerization induced by external stimuli such as pressure [29,30,31], UV irradiation [32,33], and heat treatment [28]), the corresponding polymers tend to be brown, red, or black, since the π-conjugation of the *tt* isomer is more extended than that of the *ct* isomer. Therefore, the main chain of the yellow polymers described herein (poly(**1**), poly(**3**), poly(**4**), poly(**5**), and poly(**6**)) was concluded to exhibit *ct* geometry. In view of the above, finding out whether the polymer main chain exclusively contains the *cis*-isomer is important for discussing the color differences of substituted polyacetylenes.

### 3.3. Main-Chain Structure

**Figure 3:**
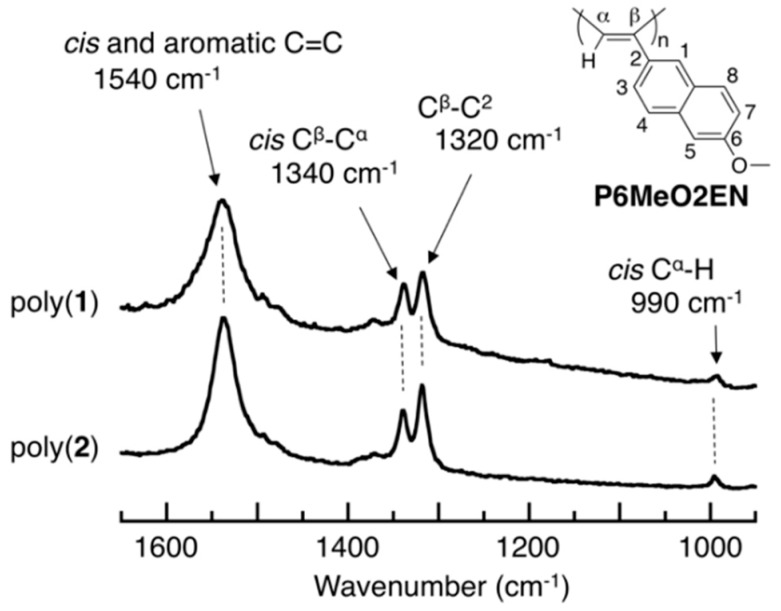
Resonance Raman spectra of P6MeO2ENs in the solid state.

The content of the *cis*-isomer in the main chain of substituted polyacetylenes is most efficiently estimated by solution-phase ^1^H NMR spectroscopy [31]. However, the insolubility of as prepared polymers in common solvents at moderate temperature forced us to use resonance Raman spectroscopy for this purpose. Figure 3 shows the spectra of poly(**1**) and poly(**2**), which had different colors despite originating from the same monomer (6MeO2EN). Both spectra exclusively featured resonance peaks attributable to the *cis*-isomer and were almost identical to that of yellow and red P2EN, which indicated that the main chains of both polymers exhibited high *cis*-isomer contents [25]. In the case of P7MeO2EN and P8MeO2EN, no peak of trans C–C bonds in the main chain (~1210 cm^–1^) was observed (Figure S3, Supplementary Materials). Thus, resonance Raman spectroscopy clearly distinguished *cis*- and *trans*-isomers in polyene chains, demonstrating that all as prepared polymers exclusively contained the *cis*-isomer. In our previous report, we showed that the absorption maximum shifted to longer wavelengths upon *cis*-to-*trans* isomerization because of the concomitant extension of π-conjugation length [31,43,44]. Therefore, the different color of P6MeO2EN implied that its helical pitch was also different from those of other polymers. The resonance peak at 1540 and 1340 cm^−1^ of poly(**2**) was somewhat sharper than that of poly(**1**), which may reflect the different bond angle distributions in the main chain of these polymers.

### 3.4. Secondary Structure

**Figure 4:**
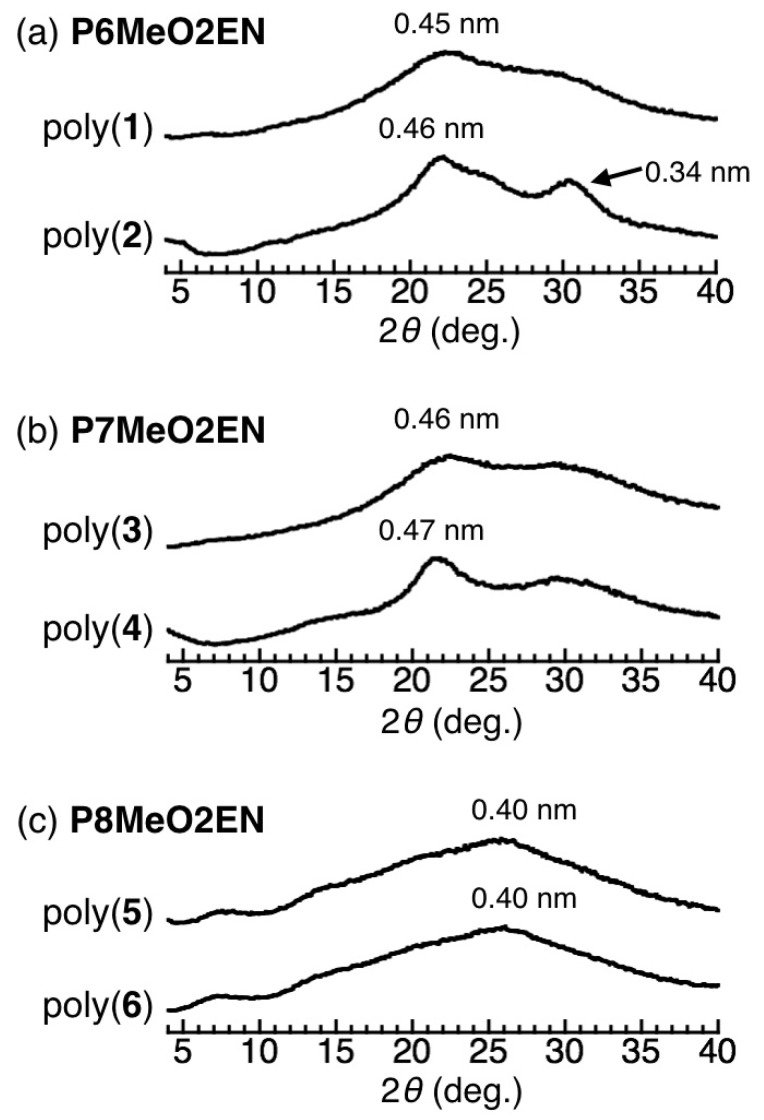
X-ray diffraction (XRD) patterns of P6MeO2ENs (**a**), P7MeO2ENs (**b**), and P8MeO2ENs (**c**).

A number of helical PPAs, P2ENs, and PPAs bearing alkoxy or alkyl groups at the *para*-position were reported to form pseudohexagonal columnar structures featuring helical polymer chains closely arranged along the same helical axis [17,45]. Consequently, the X-ray diffraction (XRD) patterns of these polymer powders exhibited (100) reflection peaks at the position corresponding to the diameter of these helices [22,23,24,25]. However, no such peaks were observed in the XRD patterns of the polymers prepared herein (Figure 4), which suggested that the persistence length of the corresponding helical chains was too short for columnar packing. Hence, we interpreted these XRD patterns assuming that all polymer chains formed helical structures without any ordered arrangement.

In the case of P6MeO2EN (Figure 4a), the pattern of poly(**1**) showed a broad peak at 0.45 nm due to an amorphous halo, whereas a distinct peak at 0.34 nm was observed only in the pattern of poly(**2**). Based on previous reports [25], this peak was concluded to reflect the distance between π-stacked neighboring naphthyl rings. Moreover, this peak allowed us to determine whether the polymer chain formed contracted helices, because the above distance was quite similar to the layer distance of graphite (0.335 nm). For this reason, we concluded that poly(**1**) and poly(**2**) comprised stretched and contracted helices, respectively.

In the case of P7MeO2EN (Figure 4b), the pattern of poly(**3**) was almost identical to that of poly(**1**), showing only a broad peak at 0.46 nm and no π-stacking-related peak. The peak at 0.47 nm observed in the case of poly(**4**) was thought to reflect the average distance between naphthyl rings. Additionally, poly(**4**) also showed a broad shoulder peak at around 30°. This peak could indicate that poly(**4**) also contained a contracted helix, although the helical pitch had a large distribution. It would also be related to the difference in 40 nm between poly(**3**) and poly(**4**) of the UV spectrum mentioned above.

In the case of P8MeO2EN (Figure 4c), the patterns of poly(**5**) and poly(**6**) were almost identical to each other and exclusively featured a broad peak at 0.40 nm, with no π-stacking-related peak observed. Thus, these patterns indicated that both polymers formed stretched helices.

### 3.5. Helical Pitch Analysis

**Figure 5:**
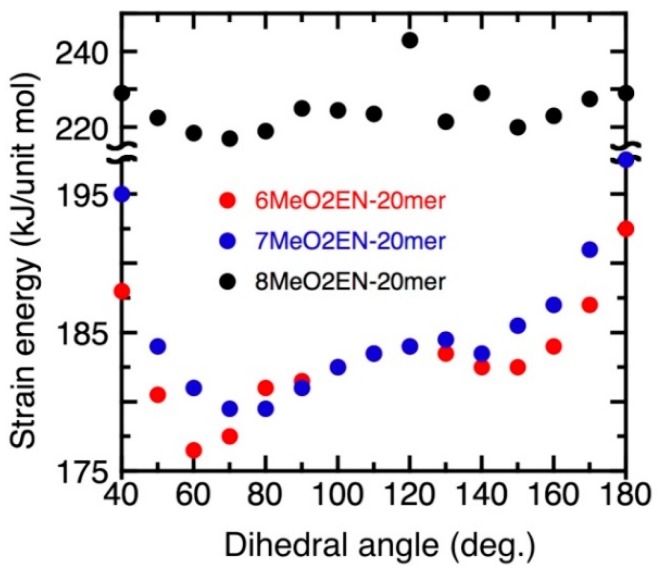
Dependencies of strain energies on the dihedral angle between two C=C bonds of C=C–C=C units for 20-mer models of 6MeO2EN, 7MeO2EN, and 8MeO2EN.

**Figure 6:**
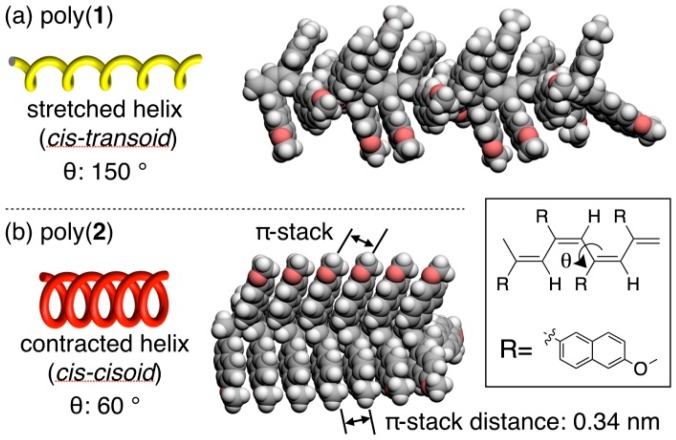
Side views of 6MeO2EN 20-mer models, showing the stretched helix of poly(**1**) (**a**) and the contracted helix of poly(**2**) (**b**).

The dihedral angles of the above helices at energetically stable conformations were analyzed by performing molecular mechanics calculations on 20-mers of each monomer as models of the corresponding polymers. The strain energy of each conformer was calculated with the initial structure in which the dihedral angle between all the monomer units in the 20-mer, that is, angles of 19 bonds for each model were changed from 180° to 40° in steps of 10° (Figure 5). P6MeO2EN and P7MeO2EN models featured global strain energy minima at 60° and 70°, respectively, which indicated that the helix of P6MeO2EN was able to contract more tightly than that of P7MeO2EN, in agreement with the results of XRD measurements. Moreover, highly ordered π-stacking with a distance of 0.34 nm between neighbor naphthyl rings was observed in the contracted helix of P6MeO2EN (θ = 60°) (Figure 6b). This stacking distance was also observed in the XRD pattern of red-colored poly(**2**) (Figure 4a). In the case of P8MeO2EN, the strain energies at all angles were much larger than those of P6MeO2EN and P7MeO2EN, which, in combination with the results of XRD analysis (Figure 4c), implied that both P8MeO2ENs probably formed stretched helices with short persistence length. Thus, we concluded that the red-colored polymer, i.e., poly(**2**), had a contracted helix, while other polymers had stretched helices.

**Figure 7:**
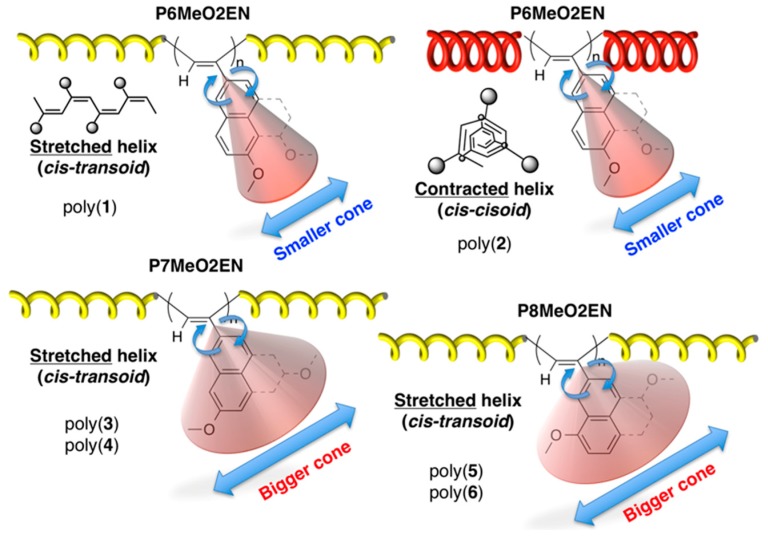
Schematic model of steric hindrances along the helical axis caused by the presence of rotatable methoxynaphthyl rings.

The contractile limit of the helical pitch depended on the position of the MeO group on the naphthyl ring. Only P6MeO2EN could form a contracted helix (poly(**2**)) when toluene was used as the polymerization solvent, whereas the use of EtOH resulted in the formation of a stretched helix (poly(**1**)), and no contracted helices could be obtained for P7MeO2EN and P8MeO2EN in both polymerization solvents. Helix contraction is expected to result in steric hindrance between the neighboring methoxynaphthyl rings because of their rotatable nature, as indicated by variable-radius cones in Figure 7. Considering the position of MeO groups, the size of the cone representing methoxynaphthyl ring rotation should be smaller for P6MeO2EN than that for P7MeO2EN and P8MeO2EN. Thus, differences in polymer color reflected those of the helical pitch. The fact that even the introduction of small MeO groups resulted in helical pitch changes implies that the introduction of other functional groups on substituted poly(arylacetylene)s should have an even larger influence.

### 3.6. DSC Study

**Figure 8:**
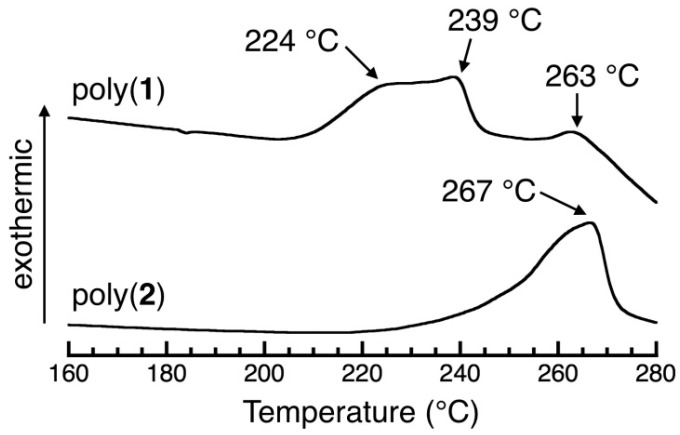
Differential scanning calorimetry (DSC) traces of P6MeO2ENs.

To determine whether the contracted helix was more thermally stable than the stretched one, we measured DSC traces of poly(**1**) and poly(**2**) (Figure 8), revealing that the trace of the former showed three exothermic peaks, while that of the latter showed only one exothermic peak. The onset temperature of the first exothermic peak was higher for poly(**2**) (220 °C) than for poly(**1**) (200 °C), which indicated that the contracted helix was more thermally stable than the stretched helix. Additionally, these temperatures were also higher than those of poly(*p*-alkoxyphenylacetylene)s (~140 °C), which indicated that large aromatic substituents increased thermal stability.

For poly(**1**), one of the two DSC peaks at 224 and 239 °C was ascribed to the *cis*-to-*trans* isomerization of the stretched *ct* helix, since these peaks were not observed in the trace of poly(**2**), with the origin of the second peak remaining unclear. The peak at ~265 °C observed in both traces was assigned to the *cis*-to-*trans* isomerization of the contracted *cc* helix. Interestingly, the presence of a small peak at 263 °C in the trace of poly(**1**) suggested that this polymer contained a small amount of the contracted helix, although it could not be detected by other measurements. The stretched-to-contracted helix conversion was thought to occur between 200 and 240 °C, similarly to the case of poly(*p*-*n*-hexoxyphenylacetylene) [22].

## 4. Conclusions

Herein, we synthesized *n*-methoxy-2-ethynylnaphthalene (*n* = 6, 7, 8) monomers and polymerized them in EtOH and toluene using an Rh-complex-based catalyst system to clarify the effect of substituent position on the helical pitch of the resulting P2ENs. Solid-state NMR, resonance Raman, and XRD measurements showed that the main chain of all prepared polymers formed a helical structure without irregular sequences. Notably, the color of the produced polymer was influenced by the polymerization solvent only in the case of P6MeO2EN. Based on the results of XRD analysis and molecular mechanics calculations, the red-colored P6MeO2EN was concluded to feature a contracted helix, whereas other polymers had stretched helices. This result indicates that, in the case of helical P6MeO2EN, the naphthyl ring MeO-substituted as position 6 exerts a relatively small rotation-induced steric hindrance compared to rings substituted at positions 7 and 8.

Thus, we concluded that the helical pitch of substituted poly(arylacetylene)s is strongly influenced by substituent position, even in the case of groups as small as MeO. Hence, the introduction of other functional groups should also influence the helical pitch, necessitating one to deeply consider the choice of substitution position. The obtained results significantly contribute to the molecular design of helical substituted polyacetylenes, since changes of the helical pitch are commonly accompanied by those of other properties. In the particular case of P2EN, introduction of functional groups at position 6 resulted in contracted helix formation, whereas the formation of a stretched helix was achieved by introducing substituents at positions 7 and 8, with the latter being more suitable for this purpose. Recently, the effect of substitution position (*o*-, *m*-, and *p*-) on the solution-phase helical pitch of PPA derivatives bearing specific amide groups was reported [35], which is important because of the potentially significant role of helical poly(arylacetylene)s in highly sensitive sensors, super-small electronic and magnetic devices, and other fields. Currently, we are working on the application of these findings to the design and synthesis of other helical poly(arylacetylene)s, and the corresponding results will be reported in due course.

## Figures and Tables

**Table 1:** Rh-catalyzed polymerization of *n*-methoxy-2-ethynylnaphthalene (nMeO2ENs). ^a^

Monomer	Polymer	Polymerization Solvent	Yield (%) ^b^	λmax(nm) ^c^
6MeO2EN	Poly(**1**)	Ethanol (EtOH)	60	440
6MeO2EN	Poly(**2**)	Toluene	74	500
7MeO2EN	Poly(**3**)	EtOH	54	440
7MeO2EN	Poly(**4**)	Toluene	91	480
8MeO2EN	Poly(**5**)	EtOH	70	450
8MeO2EN	Poly(**6**)	Toluene	98	460

^a^ Conditions: 25 °C, 0.5 h, [M]_0_ = 0.20 M, [M]_0_/[Rh cat.] = 200, [NEt_3_]/[Rh cat.] = 100. ^b^ EtOH-insoluble part. ^c^ Measured by diffuse reflectance ultraviolet–visible light (UV–Vis) spectroscopy.

## Supplementary Materials

The following are available online at http://www.mdpi.com/2073-4360/11/1/94/s1: Scheme S1: Synthesis of 7-methoxy-2-ethynylnaphthalene (7MeO2EN) and 8-methoxy-2-ethynylnaphthalene (8MeO2EN); Figure S1: ^13^C NMR spectra of nMeO2EN monomers (*n* = 6, 7, and 8); Figure S2: Room temperature ^13^C CPMAS NMR spectra of poly(**1**), poly(**3**), and poly(**5**); Figure S3: Resonance Raman spectrum of poly(**3**)~poly(**6**) in the solid state.
