# Supplementary material for: Methoxy-Group Control of Helical Pitch in Stereoregular Poly(2-ethynylmethoxynaphthalene) Prepared by Rhodium Complex Catalyst
Source: Polymers (Basel). 2019 Jan 8;11(1):94. doi: 10.3390/polym11010094 (PMC6402013; doi:10.3390/polym11010094)
Supplement: Supplementary file 1 [file polymers-11-00094-s001.pdf]

polymers

Supplementary Materials for

Methoxy-group control of helical pitch in stereoregular poly(2-ethynylmethoxynaphthalene) prepared by Rhodium complex catalyst

Yasuteru Mawatari[1,2*], Yoshiaki Yoshida[1‡], Kai Huang[3], and Masayoshi Tabata[4,5*]

[1] *Graduate School of Engineering, Muroran Institute of Technology, 27-1 Mizumoto-cho, Muroran, Hokkaido 050-8585, Japan*
[2] *Research Center for Environmentally Friendly Materials Muroran Institute of Technology, 27-1 Mizumoto-cho, Muroran, Hokkaido 050-8585, Japan*
[3] *Graphene New Materials Technology, JunFeng Business BuildingA-4F, Chongqing Road, Baoan District, Shenzhen 518101, China*
[4] *Center of Environmental Science and Disaster Mitigation for Advanced Research, Muroran Institute of Technology, 27-1 Mizumoto-cho, Muroran, Hokkaido 050-8585, Japan*
[5] *Faculty of Science and Technology, Department of Applied Chemistry and Bioscience, Chitose Institute of Science and Technology, Bibi 65-758, Hokkaido Chitose 066-8655, Japan*
* Correspondence: mawatari@mmm.muroran-it.ac.jp (Y.M.); tabata@mmm.muroran-it.ac.jp (M.T.); Tel.: +81-143-46-5964 (Y.M.); +81-143-46-5963 (M.T.)

The starting materials, 2-bromo-7-methoxynaphthalene[S1] and 2-bromo-8-methoxynaphthalene[S2] were synthesized according to the reported procedures. The monomers, 7MeO2EN and 8MeO2EN, were synthesized by using the same procedure for 2-ethynylnaphthalene (2EN) described in our previous report [25].

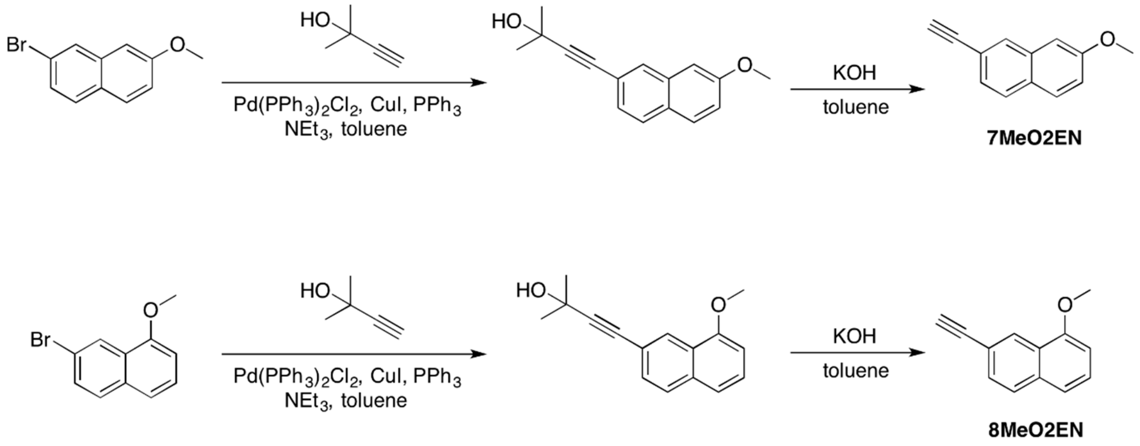

Scheme S1. Synthesis of 7-methoxy-2-ethynylnaphthalene (7MeO2EN) and 8-methoxy-2-ethynylnaphthalene (8MeO2EN).

Supplementary References

S1. Qi Zou, Xin Li, Ji Zhou, Kangkang Bai, Hans Ågren. Synthesis and photochromism of a spirooxazine derivative featuring a carbazole moiety: Fast thermal bleaching and excellent fatigue resistance. *Dyes Pigment* **2014**, *107*, 174-181.

S2. Molino, Bruce F.; Liu, Shuang; Berkowitz, Barry A.; Guzzo, Peter R.; Beck, James P.; Cohen, Marlene. Aryl- and heteroaryl- substituted tetrahydroisoquinolines and their preparation, pharmaceutical compositions and use thereof to block reuptake of norepinephrine, dopamine, and serotonin for the treatment of neurological and psychological disorders. *PCT Int. Appl.* **2006**, WO 2006020049 A2.

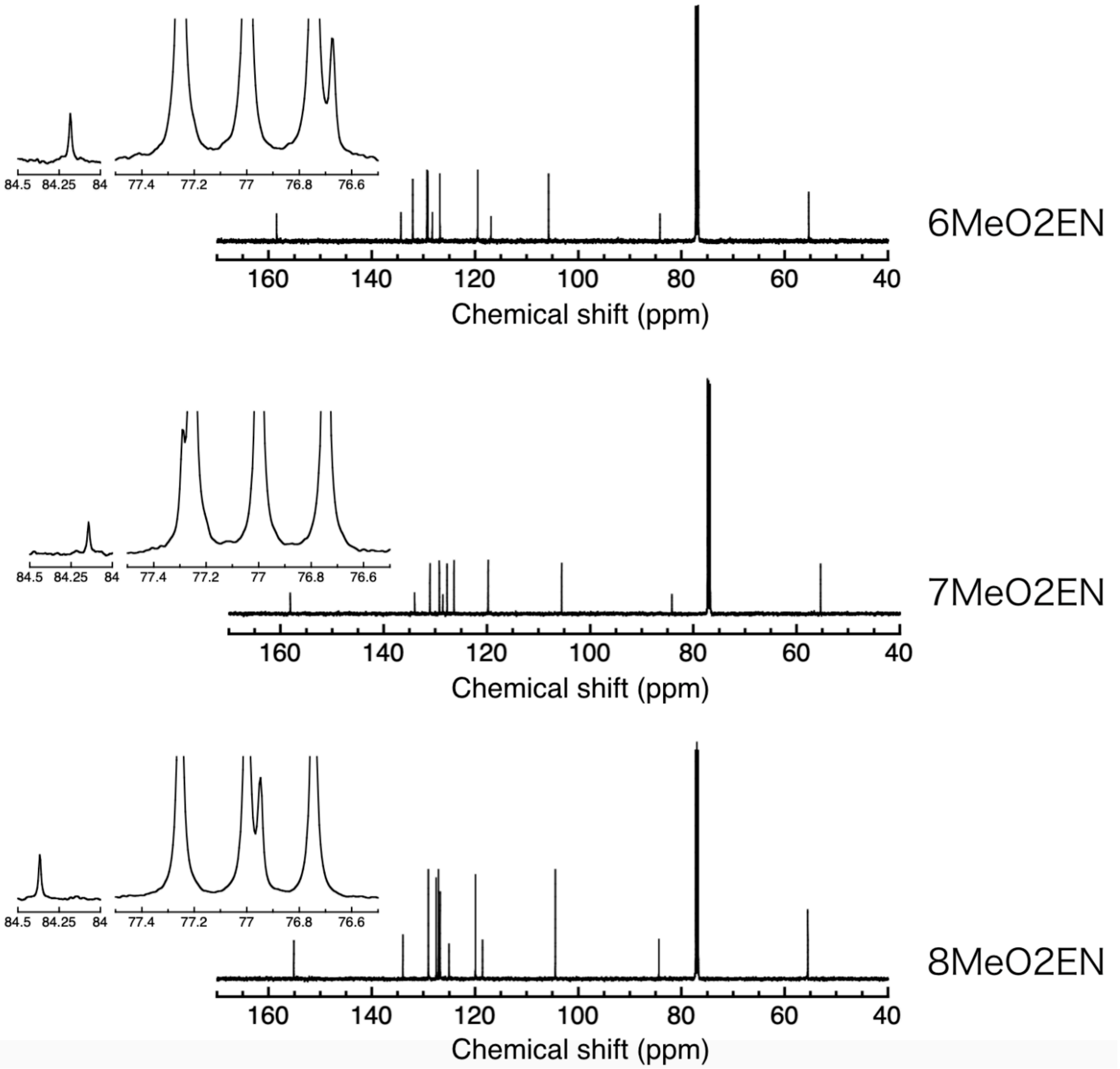

Figure S1. ^{13}C NMR spectra of nMeO2EN monomers (n=6, 7, and 8).

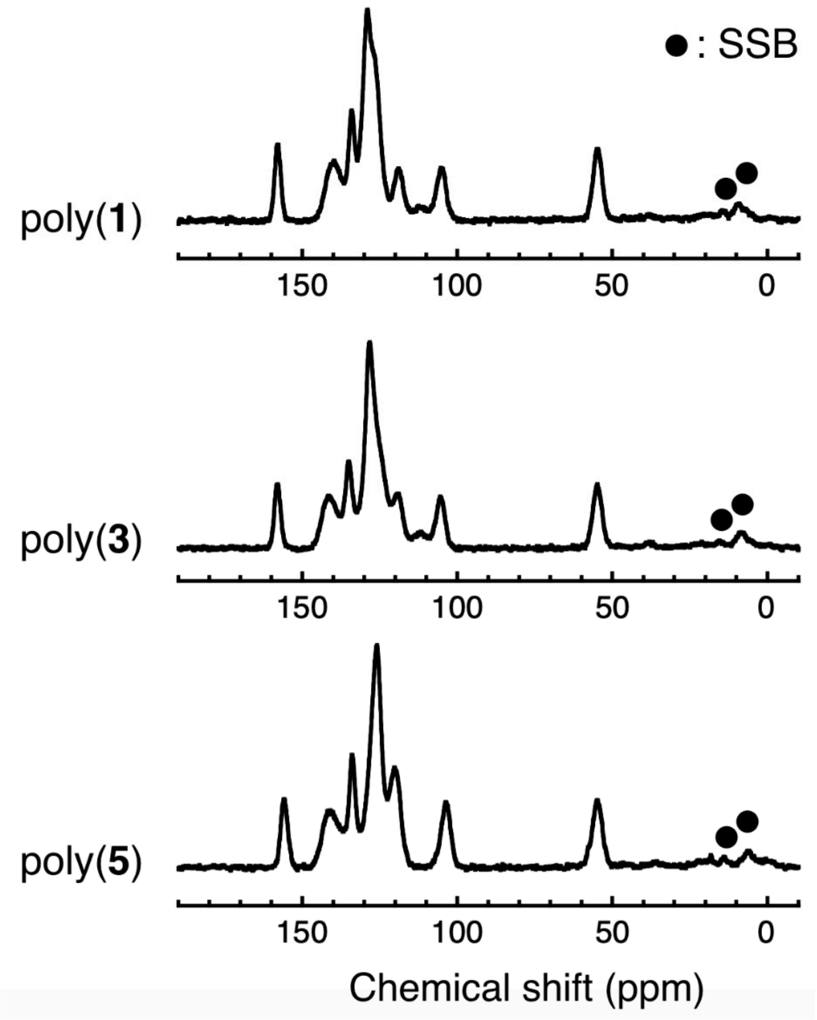

Figure S2. Room-temperature ^{13}C CPMAS NMR spectra of poly(1), poly(3), and poly(5). SSB represents spinning side band.

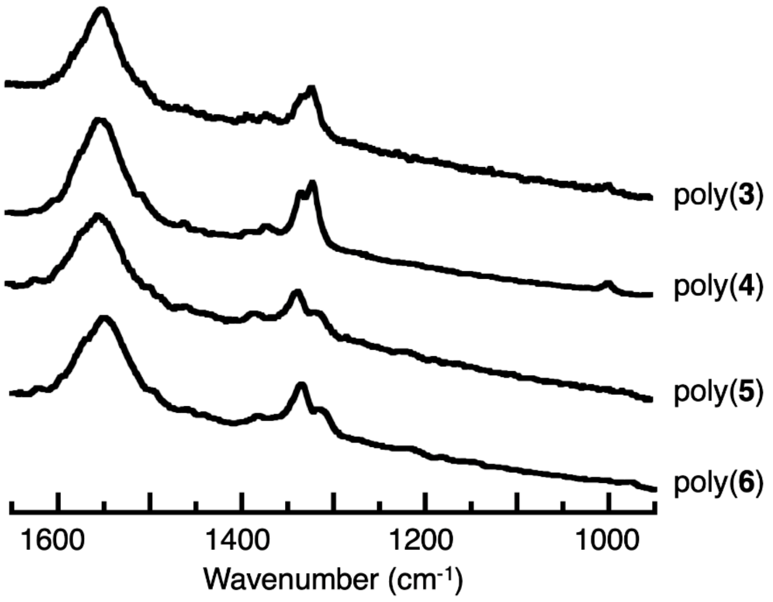

Figure S3. Resonance Raman spectrum of Resonance Raman spectra of poly(3) ~ poly(6) in the solid state.
